# Questions that this paper raises

**Published:** 2007

**Authors:** Madhukar S. Bandisode

## Response to Where is Medical Practice in India Heading? - Sunil Pandya

http://www.msmonographs.org/article.asp?issn=0973-1229;year=2006;volume= 4;issue=1;spage=50;epage=61;aulast=Pandya

Q 1: Do you agree with the author and share his pessimism? If you do not, give examples of success stories in recent Indian medical education and practice benefiting poor patients.

Ans: I agree with the author totally. I was fortunate to have studied under many teachers under whom he too had studied in the medical college. As Dr. Pandya described in his article, many of our teachers were compassionate, sincere, highly professional and considerate individuals. I totally agree with Dr. Pandya's views.

The unprofessional and unethical acts of the present day teachers and medical practitioners sadden us. I hear of the examples indicating the prevalence of greed, corruption, plagiarism and favoritism every time I visit India. It is sad that the noble profession of medicine is maligned by unscrupulous and uncouth individuals.

Q 2: The author does not consider the steps to be taken by the authorities. Can the Government, municipal corporations, associations of doctors, medical councils improve matters? If so, what steps do you suggest?

Ans: The first choice must be, ′Physician, heal thyself.′ Dr. Pandya has aptly emphasized that the ′unprofessional acts of teachers are likely to be followed by students.′ (I would say they are being followed by many of their students). The healing process should begin with:

1. Mandatory courses in medical ethics for medical students and house staff in medical colleges
2. Nationwide mandatory conferences in medical ethics for the education of practicing physicians from all branches of Medicine, including teachers in medical colleges. One physician from Mumbai approached me during one of my visits and offered to pay the US Government officials to get a visa and a Residency position in a US hospital. He could not pass the Visa Qualifying Examination. I told him to get out.
3. The basis of education in schools and colleges should be to create good citizens out of students. On the contrary, the teachers are encouraging delinquency. Unless the schools and colleges introduce a curriculum to prevent bribery, plagiarism, unprofessional behavior and selfishness, the domino effect on various professions and businesses will not end.
4. Unfortunately, regulating bodies such as Government, municipal corporations and medical councils are the propagating institutions for such practices. They will gladly develop laws and regulatory policies for punitive actions against physicians, with the resultant consequence of handing power to the administrators who in turn resort to corrupt practices. The corrupt administrators can then extract bribes from such physicians.

Q 3: What are your experiences of ethics in medical education and practice?

Ans: I have heard of bribery to get question papers prior to examinations in Mumbai. I have heard of bribing of examiners before oral and practical examinations. One official demanded money from me to clear the admission process for my relative, even when the candidate had received a letter of admission to a medical college. We turned down the admission instead of paying the official. One patient told me that he was asked to pay an exorbitant amount for an imported lens for transplantation after cataract surgery, when actually a locally manufactured lens was transplanted after receiving the money. I met one patient who was told to undergo coronary artery bypass graft (CABG) surgery a second time within a period of 3 years. He went to another cardiologist who did not find any reason for repeat CABG. I hear such accounts every time I visit India.

Q 4: There is an argument oft used by many: ‘Everyone in India is corrupt. Name one profession where there is no corruption. Why should anyone expect the medical professionals to remain free from corruption? This is the only way to survive in this country.’ Do you agree?

Ans: I do not agree with the contention. Bribery is a crime. Medical practice can be lucrative without resorting to unethical practices. The teachers in medical colleges must remain ethical. It is the responsibility of the institutions to pay them adequate remuneration. The revolution must start somewhere. The medical professionals must fight corruption and set an example for others to follow. I do not agree that this is the only way to survive in India. I am very certain that there are several teachers and practicing physicians who do not resort to corrupt practices.

Q 5. What are your views on the matter of seed and soil? Since medical colleges get as students fiercely competitive individuals who have fought tooth and nail to get in by means that are not necessarily above board, how can you expect the finished products of medical colleges to be angelic doctors?

Ans: The medical colleges have always been fiercely competitive. Did we not get into the medical colleges by competing for the seats? Did we not pass examinations without bribing? In the olden days, the students and the examiners were ethical. The reason they did not resort to such unethical acts was the culture, the ethics and respect for the profession. The current system is not only illegal, it is dangerous. It issues licenses to students who do not deserve to be in medical colleges. Those students who are below par get into medical colleges by bribing the examiners. They are not ordinary students; they are literally criminals. It is necessary to start the corrective actions as soon as possible so that the future generations of physicians will be ethical. Should the system encourage depriving admission to a deserving candidate and give it to a nondeserving criminal candidate? I think this is unjust.

Q 6: What is wrong in encouraging medical tourism? It will bring in large quantities of wealth from the Western and Arab nations, and this must inevitably benefit the country as a whole.

Ans: Medical tourism will impact several aspects of the society. The first and the most important one is that it will deprive the middle and the poor classes of deserving medical care and divert resources to the treatment of the ‘imported rich.’ Resources such as medications, equipment, space and human resources - including doctors, nurses and other hospital employees - are scarce. They will become scarcer when diverted to the ‘imported rich.’ The poor and middle class as such get lower priority than the rich in medical care. By increasing medical tourism, the disparity will increase. The non-angelic doctors of today will prefer to treat the ‘imports’ than the locals.

The riches will be distributed among those few who will sidetrack the imports for themselves or for their own institutions. They will find ways to cheat the government by pocketing the income without paying taxes. The rich will get richer and the poor will get poorer. The dream that the country will inevitably get the benefit will never be realized.

I read today a proposal for formation of an International Hospital Corporation to develop massive chain of hospitals in India. The plan calls for recruiting physicians from other countries in order to enhance the quality of care, to meet the Joint Commission for Accreditation of Hospitals (JCAHO) requirements and to collaborate with US medical institutions. This is a slap in the face of Indian medical colleges and physicians. I am proud of my Indian medical training, and I have repeatedly proven my superiority over American physicians. I know for certain the quality of education imparted by my teachers and the standard of health care provided by physicians who graduated with me and before me; they were very competent individuals.

One needs to think about the American Medical Association in this context. If the workload in USA were to decline significantly, the AMA would force Congress to halt issuing visas to non-American physicians. They will block Medicare, Medicaid and insurance payments to out-of-USA medical institutions. The whole setup in India would collapse, causing a major economic devastation. This would have a ripple effect all the way down to the poorest people. Lastly, the propensity to file lawsuits for medical errors in India would increase, which could have a devastating effect on the individuals and the institutions.

## Response to Reflections

### Turning Points in my Medical Career - Sunil K. Pandya

http://www.msmonographs.org/article.asp?issn=09731229;year= 2006; volume=4;issue=1;spage=154;epage=165;aulast=Pandya

Q 1: Are there, truly, turning points in our development where one or more striking experiences change our lives forever? Can you recall such moments in your own evolution?

Ans: Yes. There are turning points in our development. I recall several such moments in my life.

I graduated from Grant Medical College, finished internship and found out that my parents were in a major financial bind. I had to give up my plan to go in for postgraduate studies in Internal Medicine. I could not accept residency training. This was a turning point. I decided to change my goal and obtained a position in Pharmacology. The reason I chose pharmacology was (1) I found Dr. Gaitonde to be a dynamic professor and (2) pharmacology was a progressive subject and not a static one such as anatomy. I passed B.Sc., M.Sc. and M.D. in Pharmacology and supported my family. I established the Radio-Isotope Unit in the J. J. Group of Hospitals in 1963. I was the senior most lecturer in the department waiting for promotion to the Assistant Professor position in 1970.

At this juncture, Dr. Jhala, newly appointed Director of Medical Education, decided to transfer all Senior Lecturers, Assistant and Associate Professors in the whole state of Maharashtra because of a crazy belief that if Government servants were to get well rooted in one place, they would develop community contacts, which could be detrimental to the government. He transferred me to Nanded Ayurvedic College as Assistant Professor. A lecturer from B. J. Medical College, Poona, was to be transferred to G. M. C., Bombay, in my position.

Dr. Jhala did not listen to any argument. He was informed that I was trained to manage the Isotope Unit in J. J. Hospital, but he would not reconsider his order. He sacrificed the unit, which was closed shortly after I resigned. I refused to obey the order. He warned me that I would not be promoted any time in the future if I did not accept the position in Nanded. I was supposed to teach Pharmacology in Marathi to Ayurvedic students.

In a month's time, I was offered a position in a pharmaceutical company. I accepted it promptly and resigned from G. M. C. with 24 hour's notice. This was the second turning point in my career. The Dean begged me to stay but I refused, as I did not want to remain in a bureaucracy like the one that existed at that time. My goal was to remain in academic medicine. I enjoyed teaching, research, publishing papers and working in the Isotope Unit. I changed to an industrial job. I had passed the ECFMG examination in 1963, but I did not leave ‘my country’ and go to USA. I was one of those proud Indians who did not want to leave the motherland.

But an opportunity popped up again after joining the company, and I accepted a post-doctoral Fellowship in Diabetes, Endocrinology and Metabolism in the University of Alabama in Birmingham, USA. I resigned from the pharmaceutical company after 3 months of work and left India in June 1971.

It was the third turning point in my career. I was back to Internal Medicine, my original love. I was earning more and had a better life. My career changed once again - in the right direction.

Q 2: Is the author trying to be an idealist? Is it not necessary to be practical in this world? It is all very well preaching morality and goodness and the interests of the poor; but surely one has to survive, cater to the growth and development of one's own family and children. What is wrong in aspiring for riches - a mansion, Mercedes and other cars and annual holidays in countries such as Switzerland?

Ans: The author and I have experienced ideal conditions. They did exist in the past. However, control over the processes of admissions, examinations, selections for house posts, and ethical practices have been lost over a period of time. Those conditions need to be reestablished. Controls must not be brought about by punitive methods but by providing right education, inculcating responsible behavior and high ethical standards and adopting a humanitarian approach. The establishment of laws, regulations with punitive actions for failures will only breed corrupt practices.

The teachers in the olden days survived even when they took interest in the poor. One does not have to be filthy rich for the development of the family and children. I worked in government service in USA all along with a fixed salary. I do not have a million dollar mansion but just have a decent home. I had a dream to own a Mercedes Benz (MB) when Dr. Jal Patel was the Head of the Department of Pharmacology and had an MB. I bought one in USA. I realized very soon that the MB did not prove to be a good quality vehicle. It was a white elephant. I sold it and bought GM and Ford cars. They have proven more luxurious and thousand times more dependable than MB. The luxury cars of Europe did not prove their worth. Today, Americans are less attracted to European cars.

In the same way, the backwaters of Kerala attract more international tourists than European cities. Those who have become filthy rich by cheating and following unethical practices may squander their ill-gotten wealth in European cars or cities. An average Indian physician will not be enamored of such wastefulness.

Q 3: Can books ever really serve to inspire? Can you think of any books that have inspired you?

Ans: Yes, books do inspire. Have we not heard of people being carried away by ideologies presented in the books of prominent people? Holy books like the Bible, Geeta and Quran have influenced thinking of masses of people and have made martyrs out of many. Many were also influenced by the biographies of Stalin, Lenin, Abraham Lincoln and George Washington.

The works of Albert Schweitzer, his writings and his biography have made an everlasting impact on my mind too. My father treated poor patients and when they did not pay him, he never asked them again. What we observed was that when poor people suffer from illnesses and do not have the money to pay, they exhaust all home remedies and wait until the last minute to see a physician. Once treated, they will not come to the doctor until they find a way to repay him in cash or kind.

Both Schweitzer and my father survived and raised families. I was influenced by the biographies of Stalin, George Washington and Abraham Lincoln. I was also influenced by Sir Isaac Newton's biography.

Q 4: The author never refers to religion. Is he not erring gravely?

Ans: I do not know the views of the author on religion. I, for one, have spent some time reading books on comparative religion. As time passes, I feel less and less enamored by religious philosophies. It is obvious that all religions began with upheaval in their respective societies, atrocities by the rulers against the weaker sections of society or discords among the various groups in society.

The founders of religions thought about causes of disparities and recommended ways to change society. The founders of most of the religions did not live long enough to carry through their work, and the disciples changed the founder's philosophies to suit their ends. They established rituals under the garb of religion. The religions were modified over a period of time according to the needs of disciples and priests.

I do not believe Jesus Christ told his congregation to drink wine and eat bread. I do not believe Gautama Buddha told his congregation to beat on the hand-held drums before and after prayers. The original tenet of Buddha of not resorting to idol and individual's worship is forgotten. Did Hindu Gods ever tell the priest to feed them all the time? During the *pujas,* the *pujari* recites repeatedly, ′I am offering water. I am offering banana. I am offering butter. I am offering coconut. I am offering *pan.”* He spends half the time of the *puja* in trying to bribe the Gods. I am not impressed by ritualistic religions.

Religions which advocate death to widows, encourage prostitution under the garb of temple dancers, encourage caste system, deny education to women, deny freedom to women and encourage killing of other human beings because of differences in ideology/philosophy are not ethical institutions. More people in the world were killed by religious wars and conflicts than both the world wars together.

My life was not turned around by any religion. It was my own conscience that guided me throughout my life. And I believe most physicians will concur with me that their ethical behavior is not because of their religion but it is a result of their own beliefs.

## About the Author

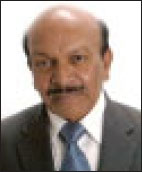



Madhukar Bandisode established the Isotope Unit in 1964 at J. J. Hospital, Mumbai. He published research papers on anthelmintics, salicylates and thyroid function. He joined the Division of Diabetes, Endocrinology and Metabolism, University of Alabama School of Medicine, Birmingham, Alabama, USA, in 1971. He provided patient care, conducted clinical and basic research in diabetes and obesity. He developed techniques for purification of various pancreatic cells. He joined the VA Medical Center, Augusta, GA, in 1975. He served as Chief, Intermediate Medicine; and Medical Director of Extended Care Services from 1979 to 2000. He retired from VAMC and now continues clinical practice in a private clinic.

